# Iliopsoas myositis ossificans after transverse myelitis in a child: a case report

**DOI:** 10.3389/fsurg.2025.1679148

**Published:** 2025-11-04

**Authors:** Haoran Yin, Jie Li, Yongtao Su

**Affiliations:** 1 The First Clinical Medical College of Shandong University of Traditional Chinese Medicine, Jinan, China; 2 Department of Pediatric Orthopedics, Affiliated Hospital of Shandong University of Traditional Chinese Medicine, Jinan, China; 3 Shandong University of Traditional Chinese Medicine, Jinan, China

**Keywords:** neurogenic heterotopic ossification, iliopsoas, transverse myelitis, pediatric, surgical procedure

## Abstract

**Introduction:**

Acute transverse myelitis in children is a rapidly progressive disease of unknown etiology, with some patients experiencing lifelong paralysis. Myositis ossificans (MO) refers to heterotopic ossification within soft tissues. Neurogenic MO is rare in clinical practice and is often misdiagnosed, particularly in pediatric patients who are unable to clearly describe their symptoms. Diagnosis typically relies on imaging studies.

**Methods:**

This report describes a case of an 8-year-old child who developed paraplegia following a diagnosis of acute transverse myelitis and subsequently presented with MO of the iliopsoas muscle, likely resulting from the spinal cord injury in combination with a distal femoral fracture, although the ossification itself developed in the iliopsoas region rather than at the fracture site.

**Results:**

The patient exhibited progressive limitation of hip joint movement during rehabilitation. X-ray imaging revealed high-density ossification in the iliopsoas region. After six months, the ossification had matured extensively, and surgical resection was performed. The patient's hip mobility improved postoperatively. During a 3-year follow-up period, there was no recurrence of ossification.

**Discussion:**

This case highlights the potential development of neurogenic MO in pediatric patients with spinal cord injury, even when the ossification does not occur at the fracture site. We discuss the challenges in diagnosing and treating such conditions in children and review current diagnostic and therapeutic advancements. This case contributes to the understanding of neurogenic MO in pediatric patients and provides insights for managing similar conditions.

## Introduction

Myositis ossificans (MO) refers to heterotopic ossification (HO) occurring in soft tissues such as muscles, tendons, and ligaments. Based on etiology, MO can be classified into progressive MO (also known as fibrodysplasia ossificans progressiva, FOP) and non-hereditary MO (1). Neurogenic MO, particularly following spinal cord injury, is a rare subtype of the latter and can be easily misdiagnosed in clinical practice. Here, we report a pediatric case of iliopsoas MO secondary to paraplegia from transverse myelitis, with additional predisposing factors including a distal femoral fracture, although the ossification developed in the iliopsoas region, and we provide a clinical analysis and summary of its diagnostic and therapeutic management.

## Case presentation

In January 2020, an 8-year-old child presented with high fever of unknown cause and was treated symptomatically with antipyretics. Several days later, the child experienced sudden onset of lower limb paralysis. Further evaluation at a local hospital led to the diagnosis of transverse myelitis. Despite treatment with high-dose corticosteroids and neurotrophic agents, the therapeutic response was poor. Corticosteroid administration induced secondary osteoporosis, and even minor physical activity resulted in a right distal femoral fracture. The patient underwent open reduction and internal fixation (ORIF) for a distal femoral fracture.

**Figure 1:**
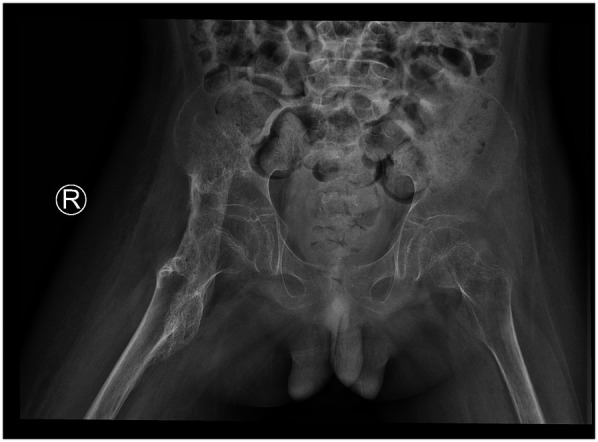
2020.09. A high-density ossification with blurred edges and cross-joint growth can be seen in the iliopsoas muscle area of the right hip, indicating that it is not yet fully mature.

During rehabilitation in September 2020, significant restriction of right hip motion was noted. Pelvic x-ray revealed a high-density ossified mass along the course of the right iliopsoas muscle, with blurred margins and extension across the hip joint (Figure 1). As the ossification was presumed to be in the early to intermediate stage and not yet fully matured, conservative management including adjustment of rehabilitation exercises to avoid excessive mechanical stress, and close radiographic follow-up was recommended. Pelvic x-rays were obtained every 4–6 weeks to monitor lesion progression, with assessment of density, margin definition, and trabecular development to determine the appropriate timing for surgical resection.

Differential Diagnosis: Several differential diagnoses were considered before confirming neurogenic MO of the iliopsoas. FOP (2): Systemic hereditary disorder with congenital great-toe malformations and progressive, symmetric ossification. Our patient lacked typical skeletal anomalies and showed unilateral, localized ossification only; genetic testing for ACVR1 mutation was not performed because clinical criteria for FOP were not met. Abscess/Infection, Imaging studies show mature trabecular bone rather than necrotic or solid enhancing tissue. Soft-tissue sarcoma, imaging examination showed no invasive periosteal reaction and clear boundaries. Tumoral calcinosis, typically presents as a periarticular cystic calcified mass that spares the synovium (3). Fine-needle aspiration reveals chalky fluid. In this case, the lesion was an intramuscular solid lesion that demonstrated a cortical/trabecular architecture. Iliacus hematoma, Al et al. (4) recently described an iliacus hematoma presenting 3 weeks after minor trauma and causing late femoral-nerve palsy. In contrast, our patient's lesion demonstrated progressive ossification with cancellous bone formation on CT, and radiographic evolution clearly distinguished it from hematoma. Taken together, the unilateral location, history of spinal cord injury plus fracture, zonal maturation on sequential imaging, and benign histology confirmed the diagnosis of neurogenic MO and excluded the above conditions.

**Figure 2:**
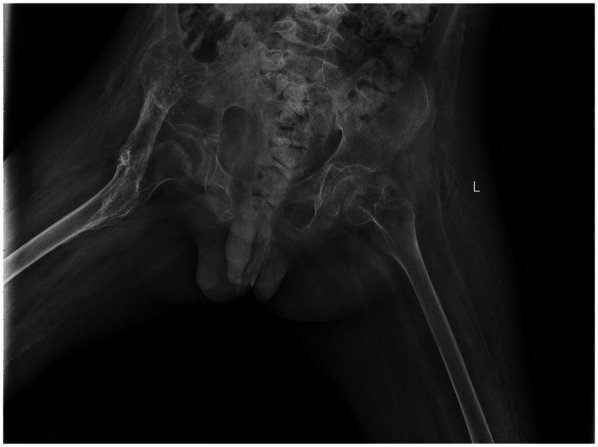
2021.04. A strip-like high-density ossification shadow is consistent with the long axis of the iliopsoas muscle, with clear edges, growing across the joint and surrounding the joint.

By April 2021, repeat radiographs showed a well-demarcated, band-like high-density ossification consistent with the anatomical course of the iliopsoas muscle, the cortical shell is ≥1 mm and continuous (Figure 2). The mass extended across the hip joint and partially encased it. After excluding surgical contraindications, surgical excision was performed under general anesthesia. The child is in the supine position; a 12 cm L-shaped anterior incision was made over the right hip. After incising the skin, careful blunt dissection was performed; the soft tissues were used to retract the femoral nerve and artery medially, exposing the HO. The HO was separated proximally to the anterior superior iliac spine and distally to the area above the lesser trochanter. The mass was transected at both ends using surgical instruments, and the surrounding soft tissues were carefully detached. The ossified segment was successfully excised. Bone rongeurs were used to smooth the bony edges. Intraoperative assessment revealed that hip flexion reached 90°. Fluoroscopy confirmed complete removal of the HO. Bone wax was applied to control bleeding, and the incision was closed in layers. The amount of bleeding was about 350 ml, and red blood cells were transfused to replenish blood volume. A negative pressure drainage tube was left in place after the operation and was removed after 48 h when no obvious drainage fluid was found.

**Figure 3:**
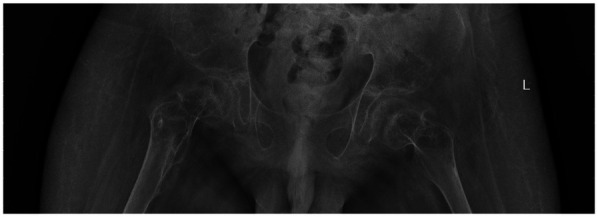
2021.12. Eight months after surgery, no recurrence of heterotopic ossification was observed.

Postoperatively, the wound healed well, and the patient was discharged with instructions for continued functional rehabilitation. The patient received indomethacin (dosage calculated according to weight) after surgery to reduce the risk of recurrence. Liver and kidney function were monitored during treatment. Follow-up 6 weeks after surgery showed no new ossification, and the drug was discontinued. Follow-up at regular intervals demonstrated no recurrence (Figure 3).

As of June 2024, the patient's hip joint function remained satisfactory. Due to lower body paraplegia, the patient's passive flexion range was approximately 0°–85°, extension range was approximately 0°–5°, and abduction range was approximately 0°–25°, allowing for wheelchair use. Follow-up imaging studies revealed no signs of recurrence of ossification, and the patient and family expressed satisfaction with the treatment outcome. For timeline and management decisions, please refer to Tables 1, 2.

**Table 1:** Timeline.

Time	Event
2020.01	High fever, sudden paralysis of both lower limbs ➜ Diagnosed as “acute transverse myelitis” by the local hospital
2020.02	Osteoporosis after hormone shock, right distal femoral fracture caused by minor trauma, right distal femur ORIF surgery was performed.
2020.09	During rehabilitation training, limited right hip flexion was found. Pelvic x-ray first revealed cloud-like heterotopic ossification in the iliopsoas region.
2020.09–2021.04	Follow-up imaging every 4–6 weeks showed that ossification gradually matured and the border became clear.
2021.04	Ossification matured, surgical resection was performed (12 cm bone bridge).
2021.05–2024.06	Regular rehabilitation, no recurrence; last follow-up 2024-06, hip range of motion was satisfactory.

**Table 2:** Management decisions.

Time	Stage	X-ray	Management decisions
2020.09	Early-middle period	A high-density ossification with blurred edges and cross-joint growth can be seen in the iliopsoas muscle area of the right hip, indicating that it is not yet fully mature.	Adjustment of rehabilitation exercises, close radiographic follow-up
2021.04	Maturity	A strip-like high-density ossification shadow is consistent with the long axis of the iliopsoas muscle, with clear edges, growing across the joint and surrounding the joint.	After evaluation, surgical resection of heterotopic ossification tissue was performed.

Because the diagnosis of MO relies primarily on imaging features and histopathological findings, systemic laboratory parameters (such as inflammatory markers or serum calcium and phosphate levels) were not routinely collected in this study. This patient showed no signs of systemic inflammation or metabolic abnormalities.

## Discussion

It has been reported that approximately 10%–53% of patients with central nervous system injuries develop HO (5). Risk factors include the severity of the injury and the neurological level of spinal cord involvement. In patients with spinal cord injury, HO typically forms caudal to the lesion level and most frequently affects the hip joints. In contrast, patients with traumatic brain injury may develop HO at multiple anatomical sites, including the hips, knees, elbows, and shoulders (6).

Radiologically, HO evolves dynamically, and its imaging features vary across the early, intermediate, and late stages. In the early stage, it typically presents as soft tissue swelling with ill-defined margins and cloud-like or irregular high-density opacities. The intermediate stage is characterized by patchy, strip-like, or eggshell-like calcifications. In the late stage, large sheet-like or band-shaped high-density lesions become apparent, with clearly defined cortical margins and visible internal trabecular bone structures (7).

Current early-stage intervention strategies for HO include NSAIDs, bisphosphonates, and radiotherapy. Short-term NSAIDs are used to inhibit the recurrence of HO. The dosage should be calculated based on the patient's weight. Patients should be re-evaluated in the outpatient clinic after 6 weeks of continuous use. If no new ossification is found on imaging examinations, the drug can be discontinued. During medication, monitor liver and kidney function, electrolytes, and gastrointestinal symptoms. Once HO develops, bisphosphonates are the intervention with the strongest supporting evidence (5). However, there are only case series in pediatrics and no RCTs, so it can only be used as an auxiliary when NSAIDs are contraindicated or insufficient. Radiotherapy is effective in preventing HO in adults but is generally not recommended for children (8). Therefore, early intervention strategies should be tailored to each patient's clinical condition and individual risk factors.

When non-surgical interventions are ineffective and the condition significantly impairs the patient's function, surgical resection should be considered. However, surgical timing is critical and should correspond to the maturity of the ossified mass. It is generally recommended that: Trauma-related HO be resected after 6–9 months, Spinal cord injury-associated HO after approximately 12 months, and Traumatic brain injury-associated HO after around 18 months (9).In this case, the patient was a young child who developed spinal cord injury secondary to acute transverse myelitis—a significant risk factor for HO involving the hip region. Additionally, corticosteroid-induced osteoporosis and subsequent fragility fracture may have triggered bone remodeling pathways. Inappropriate or excessive rehabilitation exercises may also have contributed to the pathogenesis of iliopsoas HO. During rehabilitation, progressive restriction in hip range of motion was noted. Imaging studies revealed immature HO. The patient was managed conservatively with close follow-up and NSAID therapy. Once imaging confirmed mature ossification, surgical resection was performed to improve the patient's quality of life.

This study has certain limitations. Because the patient's imaging studies showed a clear peripheral mature ossification zone and the family declined further pathological examination, the patient's pathological findings are not reported. Meanwhile, the absence of systematic laboratory data, which could have provided supportive information for differential diagnosis and follow-up monitoring.

### Parent perspective

We were very anxious when our child's hip range of motion gradually deteriorated during recovery. After surgery, we saw significant improvements in hip range of motion and ease of daily care. We'd like to share our experience to help other families recognize this complication earlier.

## Conclusion

A thorough assessment of risk factors for HO is essential in the clinical management of patients with neurological injuries. Early prevention, prompt identification, accurate diagnosis, and appropriate treatment are critical for minimizing functional impairment caused by HO. In this case, spinal cord injury secondary to transverse myelitis was a major predisposing factor for MO. However, corticosteroid-induced osteoporosis and a distal femoral fracture may also have contributed to the pathological environment that facilitated MO formation. Management strategies should be individualized, based on the patient's clinical presentation and progression. Furthermore, ongoing research into the molecular mechanisms underlying HO formation is necessary to identify effective therapeutic and preventive targets.

## Data Availability

The original contributions presented in the study are included in the article/Supplementary Material, further inquiries can be directed to the corresponding authors.
